# Depression, anxiety, and medication adherence among Sudanese refugees with chronic illnesses in Aftit refugee camp, northwest Ethiopia: a cross-sectional study

**DOI:** 10.1080/16549716.2025.2573509

**Published:** 2025-10-29

**Authors:** Alemante Tafese Beyna, Habtamu Semagne Ayele, Abaynesh Fentahun Bekalu, Abebech Tewabe Gelaye, Demis Getachew, Assefa Kebad Mengesha

**Affiliations:** a Department of Pharmacology, School of Pharmacy, College of Medicine and Health Sciences, University of Gondar, Gondar, Ethiopia; b Department of Clinical Pharmacy, School of Pharmacy, College of Medicine and Health Sciences, University of Gondar, Gondar, Ethiopia

**Keywords:** Refugee, anxiety, depression, adherence, and mental health

## Abstract

**Background:**

Anxiety and depression are common among refugees and can affect medication adherence. However, no prior study has evaluated the mental health status and medication adherence of Sudanese refugees with chronic illness in Aftit refugee camp, Ethiopia.

**Objective:**

To assess the prevalence of anxiety, depression, and medication adherence among Sudanese refugees in Aftit refugee camp, Ethiopia.

**Method:**

A cross-sectional study was conducted from October 1–30, 2024, 231 Sudanese refugees with chronic illnesses were randomly selected. Bivariate and multivariate logistic regression analyses were used. A *p*-value < 0.05 was considered statistically significant.

**Results:**

The response rate was 96.25%. The prevalence of depression and anxiety symptoms, assessed using the Hopkins Symptom Checklist, was 48.1% and 48.5%, respectively, while 42% of participants demonstrated good medication adherence based on a 6-item adherence tool. Depression was significantly associated with living alone (AOR = 6.50), longer camp stay (≥1 year) (AOR = 3.34), food/water shortages (AOR = 3.17), Khat use (AOR = 2.46), and inadequate shelter/clothing (AOR = 4.63). Anxiety was significantly associated with age > 60 (AOR = 2.98), being divorced (AOR = 5.67) or widowed (AOR = 8.36), Khat use (AOR = 2.55), loss of family/friends due to violence (AOR = 9.68), torture (AOR = 3.37), and imprisonment (AOR = 2.42). Medication adherence was significantly associated with aged > 60 (AOR = 4.78), those with higher education (AOR = 4.88), and those living alone (AOR = 3.33).

**Conclusion:**

The study underscores the importance of integrating mental health screening into refugee care and addressing structural challenges like housing, and substance use to improve both mental health and medication adherence. These findings can inform health policies and refugee programs by identifying high-risk groups and guiding targeted interventions.

## Paper Context

- **Main findings:** Nearly half of the Sudanese refugees with chronic diseases experienced anxiety and depression, and only 42% had good medication adherence.
- **Added knowledge:** This study provides the first evidence from Ethiopia linking mental health conditions with medication adherence among Sudanese refugees with chronic illnesses.
- **Global health impact for policy and action:** The findings emphasize the urgent need to integrate mental health services into chronic disease care in refugee settings and to address basic living conditions such as shelter, food, and trauma support.

## Background

Mental health is a state of well-being that allows individuals to recognize their potential, manage everyday stress, work effectively, and actively participate in their communities [1]. Among mental health disorders, depression and anxiety are the most prevalent globally, affecting hundreds of millions of people [2]. Anxiety, characterized by persistent worry and fear, can manifest in excessive stress, panic attacks, or phobias. Globally, anxiety disorders affected 359.2 million people in 2021 [3]. Depression, marked by persistent sadness, lack of interest, and feelings of hopelessness, affects daily functioning and can lead to severe consequences like suicidal ideation [4]. Approximately 332.4 million people worldwide experience depression [3]. Experiencing violence, armed conflict, forced displacement, and social or economic instability like the ongoing conflict in Sudan greatly increases the risk of depression and anxiety, as these factors disrupt daily routines, create uncertainty, and reduce access to essential resources [5].

In Sudan, ongoing internal conflicts and recent clashes between armed groups have created widespread violence and instability, more recently, on 15 April 2023, violent clashes broke out between the Sudanese Armed Forces (SAF) and the paramilitary Rapid Support Forces (RSF) [6]. This ongoing war has caused profound hardship for the Sudanese people. Many families have lost their loved ones because of violence, and broken healthcare services mean many people cannot get the medical care they need. The tough conditions of being forced to move, living in poverty, and trying to start over have left many people in tough situations [7].

As a result of the violence, many Sudanese have been forced to leave their homes, looking for safety inside Sudan or in nearby countries like Ethiopia [8]. Among these displaced people, those who have chronic illnesses in refugee camps face tough challenges. Disrupted healthcare systems and lack of resources make it hard for them to get the medicine they need or follow their treatment plans, making their health even worse [9–11]. Also, the stress of having to move has caused many mental health problems, like anxiety and depression, making the challenges for refugees even harder [12].

Refugee populations are disproportionately affected by these mental health disorders, especially those caused by the trauma of war and displacement [13]. Research shows that after disasters, psychological symptoms can significantly impact individuals and their communities, with around 10% of trauma-affected populations developing serious mental health conditions and another 10% exhibiting severe behavioral difficulties [14,15]. These challenges are particularly severe among refugees with chronic illnesses, as managing both physical and mental health becomes increasingly difficult without proper healthcare [16].

Moreover, anxiety and depression can hinder individuals from adhering to their medication regimens, further complicating the management of chronic conditions [17]. Medication adherence is essential for managing chronic illnesses and ensuring better health outcomes [18]. However, refugees face unique challenges that hinder adherence, including language barriers, cultural differences, and logistical obstacles like transportation and medication affordability [10,19]. Mental health conditions exacerbate these challenges; depression can lead to forgetfulness and lack of motivation, while anxiety may cause avoidance behaviors, both of which disrupt consistent treatment [20].

Despite the large number of refugees in low-income countries, most research on refugee mental health has focused on those in Western nations [21]. There is currently limited data on the prevalence of depression, anxiety, and medication adherence among participants in the Aftit refugee camp, in northwest Ethiopia. This aim of this study was to assessed anxiety, depression, and medication adherence among Sudanese refugees with chronic illness in the Aftit Refugee Camp, Ethiopia.

This research provides crucial insights into the mental health challenges faced by refugees, particularly in Ethiopia, and highlights the unique struggles of managing chronic illnesses in resource-limited settings. The findings can guide policymakers and aid organizations in designing more effective support systems and targeted interventions. Additionally, this study offers practical implications for mental health professionals, counselors, and aid workers, equipping them to better address the specific needs of Sudanese refugees.

## Method

### Study design and setting

This cross-sectional study was carried out among Sudanese refugees with chronic disorders residing in the Aftit refugee camp over the period of October 1–30, 2024. The camp is situated near the border with Sudan, close to Metema, a town in the West Gondar administrative zone of the Amhara National Regional State, approximately 897 kilometers northwest of Ethiopia’s capital, Addis Ababa. This new site was opened after the closure of two other sites Awlala and Kumer, due to security issues. Established in August 2024, the camp was set up by Ethiopia’s Refugees and Returnees Service (RRS) and the United Nations High Commissioner for Refugees (UNHCR) to accommodate individuals fleeing the conflict in Sudan.

### Source and study population

The source population for this study comprised Sudanese refugees living in the Aftit refugee camp who were taking medication for chronic disorders. Because the study aimed to examine the long-term impact of chronic conditions on mental health and medication adherence in refugee’s population, refugees with only short-term or acute illnesses were not included. The study population therefore consisted of individuals who were on medication for chronic conditions who were residing in the camp during the data collection period.

### Inclusion and exclusion criteria

For this study, we included Sudanese refugees who were 18 years or older and living in the Aftit refugee camp. Participants had to have been taking medication for chronic conditions for at least three months before the study period were eligible to participate. Only those who were willing and able to provide informed consent could take part in the study.

We excluded refugees who had communication difficulties that would make it hard for them to participate in interviews. We also excluded refugees with cognitive impairments due to mental illness or other conditions that might affect their understanding or participation in the study.

### Sample size calculation and sampling technique

To determine the sample size, the single population proportion formula was applied.

$$n=\frac{{\left(\mathrm{Z\alpha}/2\right)}^{2}\times P\left(1-p\right)}{d^{2}}$$

$$n=\frac{{\left(1.96\right)}^{2}\times 0.5\left(1-0.5\right)}{0.05^{2}}$$

$$n=\frac{3.84\times 0.5\left(0.5\right)}{0.0025}\;=\;384$$

P: Proportion in the target population, There is no reasonable estimate since no prior study has been conducted in the Ethiopian setting to assess mental health and medication adherence among refugee patients with chronic illnesses. Therefore, we used 50% (i.e. 0.5) to maximize the sample size. *n* = calculated sample size, (α = 0.05), 95% confidence interval (Z α/2 = 1.96), and absolute precision or margin of error, 5% (d = 0.05). The total sample size for Sudan refugees with chronic illness was 384. But the total Sudan refugees with chronic illness population at Aftit was estimated between (N) = 400–500 which is <10,000. Therefore, we used the finite population correction formula to determine the final sample size.

Finite population correction formula:

Where:

$$\mathrm{nf}=\frac{\mathrm{no}}{1+\frac{n0-1}{N}}$$

$$\mathrm{nf}=\frac{384}{1+\frac{384-1}{500}}\;=\;217.44$$

Where:

nf = Adjusted sample size for a finite population

no = Initial sample size from an infinite population calculation

*N* = Total population size

By adding a 10% non-response rate of 218 × 10% = 21.8, the final sample size was 240.

Participants were selected using a simple random sampling method. Data collection occurred on chronic illness follow-up days, specifically on Tuesday and Friday, for one month, from October 1–30, 2024. On each follow-up day, approximately 30 patients were chosen. Each eligible patient was assigned a unique identifier, and a random number generator was utilized to ensure that every individual had an equal chance of being included

### Variable

#### Dependent variables

The dependent variable of this study were medication adherence, Depression, and Anxiety symptoms.

#### Independent variable

The study considers a variety of factors as independent variables. Sociodemographic characteristics cover details like age, gender, marital status, educational level, living conditions, and number of family. Behavioral factors focus on habits such as Khat chewing, cigarette smoking, and how participants coped with shortages of food or water. The study also considers traumatic experiences, including the loss of family or friends to murder, imprisonment, torture, or struggles with inadequate housing or clothing. Clinical characteristics types of chronic conditions such as cardiovascular, endocrine, psychiatric, infectious diseases, and the number of medications prescribed.

### Data collection tools and procedures

Data were collected using a structured questionnaire adapted from previously published studies [22–25]. Data were collected through an interviewer-administered questionnaire by two trained BSc nurses using the Kobo Toolbox mobile application.

### Assessment of anxiety and depression

Participant’s Anxiety and depression level was assessed using Hopkins Symptom Checklist (HSCL-25). Permission to use the HSCL-25 was obtained through a copyright license prior to the start of the study. The HSCL-25 includes 25 questions, with the first 10 focusing on anxiety and the remaining 15 addressing depression. Responses were recorded on a 4-point Likert scale: ‘Not at all’ (1), ‘A little’ (2), ‘Quite a bit’ (3), and ‘Extremely’ (4). The depression score was calculated as the average of the 15 depression-related items, while the anxiety score was derived from the average of the 10 anxiety-related items. The study used the Arabic version of the scale, which has already been tested and shown to be reliable and valid in previous research [23]. It has also been validated in Africa [26] and is widely used among displaced and refugee populations in sub-Saharan countries like Ethiopia, Kenya, Uganda, and Sudan [27,28].

### Assessment of medication adherence

Medication adherence was assessed using a 6-item tool, with responses recorded on a binary (Yes/No) scale. A ‘Yes’ response was assigned a score of 1, and a ‘No’ response was assigned 0. The total adherence score ranged from 0 to 6, calculated by summing the individual scores [24]. These scores were then converted to a 0–100 scale, with an 80% threshold used for classification. Patients scoring ≤1 were categorized as ‘adherent,’ while those scoring ≥2 were classified as ‘non-adherent’, based on previous studies [25].

### Data quality control

The questionnaire was first developed in English and then translated into Arabic, the local language of Sudanese, by native speakers. Intensive training was provided to the data collectors and supervisors to ensure they were well-prepared for their roles. The training covered the data collection process, study goals, and made sure everyone understood the terms and tools used. It also highlighted the importance of quickly organizing and submitting the collected data. To ensure everything was consistent, a pretest was done with 5% (12) of participants, who were then excluded from the final study. Based on the feedback, we made some changes, including rewording and improving the data collection tool.

### Operational definitions

*Adherent*: If the adherence score is ≤1 [24,25].

*Non-adherent*: If the adherence score is ≥2 [24,25].

*Depressive symptoms*: If the mean score on the depression subscale of HSCL-25 is ≥1.75 [22,23].

*No depressive symptoms*: If the mean score on the depression subscale of HSCL-25 is <1.75 [22,23].

*Anxiety symptoms*: If the mean score on the anxiety subscale of HSCL-25 is ≥1.75 [22,23].

*No anxiety symptoms*: If the mean score on the anxiety subscale of HSCL-25 is <1.75 [22,23].

*Chronic disease*: A chronic disorder is a long-lasting medical condition that persists for a year or more and requires ongoing treatment or management. In this study, chronic disorders include respiratory diseases such as asthma, chronic obstructive pulmonary disease (COPD), and interstitial lung disease (ILD); cardiovascular conditions including hypertension, heart failure, and coronary artery disease (CAD); endocrine disorders such as diabetes and thyroid disorders; neurological conditions including epilepsy and Parkinson’s disease; psychiatric disorders such as depression, anxiety, and schizophrenia; infectious diseases including HIV and hepatitis; and other illnesses such as cancer, chronic kidney disease (CKD), and liver cirrhosis.

### Data entry and statistical analysis

The data were analyzed using SPSS version 25.0 and presented as frequencies and percentages in descriptive texts, tables, and figures. To explore the association between dependent and independent variable both bivariate and multivariate logistic regression were used. Variables with a p-value ≤0.2 in the bivariate analysis were included in the multivariate analysis. In the multivariable binary logistic regression, a *p*-value < 0.05 was used to determine statistical significance, the adjusted odds ratio measured the strength of the association, and the Hosmer-Lemeshow test assessed how well the model fit the data. The reliability of the questionnaire was tested using Cronbach’s alpha, which showed an excellent reliability score of 0.914.

### Ethical considerations

The University of Gondar, Institutional Research Review Board of the College of Medicine and Health Sciences and Specialized Hospital provided official approval for the study with the protocol number R/T/T/C/Eng./013/9/2024. To uphold ethical standards, the study adhered to the principles outlined in the Declaration of Helsinki. Participants were informed about the confidentiality of their data, and written consent was obtained to confirm their voluntary participation. They were also given the freedom to withdraw from the study at any time.

## Result

### Socio-demographic characteristics, clinical characteristic, and medication use of participants

Of the 240 participants, 231 completed the questionnaire, resulting in a response rate of 96.25%. The remaining nine were excluded due to refusal (*n* = 5) or incomplete responses (*n* = 4).The majority of participants were aged between 40 to 60 years (102, 44.2%). A significant proportion were male (131, 56.7%) and lived with family (141, 61.0%). The most common chronic condition was respiratory disorders (57, 24.7%). Nearly half of the participants (113, 48.9%) were prescribed four or more medications (Table 1).

**Table 1.** Socio-demographic characteristics, clinical characteristics, and medication use among Sudanese refugees with chronic illnesses in Aftit refugee camp, Ethiopia, 2024 (*N* = 231).

Variables	Category	Frequency (%)
Age	<40	74 (32.0)
	40–60	102 (44.2)
	>60	55 (23.8)
Gender	Male	131 (56.7)
	Female	100 (43.2)
Marital status	Single	45 (19.5)
	Married	107 (46.3)
	Divorced	60 (26.0)
	Widowed	19 (8.2)
Educational status	Unable to read and write	57 (24.7)
	Elementary school	62 (26.8)
	High school	49 (21.2)
	Higher institution	63 (27.3)
Living condition	With family	141 (61.0)
	Alone	90 (39.0)
Number of family	1	50 (21.6)
	2–4	64 (27.7)
	>4	117 (50.6)
Types of chronic condition	Resparitory (astma, COPD, Ild)	57 (24.7)
	Cardiovascular (Htn, HF, CAD)	32 (13.9)
	Endocrine (DM, thyroide disorder)	30 (13.0)
	Neurological (Epilepsy, Parkinson)	23 (10.0)
	Psychatry condition (Depression, Anxity, psychophernia)	53 (22.9)
	Infectiose (HIV, hepatites)	25 (10.8)
	Other (onchology, CKD, cirossise … .)	11 (4.8)
Number of prescribed medication	1	46 (19.9)
	2–3	72 (31.2)
	≥4	113 (48.9)

Abbreviation: COPD (Chronic Obstructive Pulmonary Disease), ILD (Interstitial Lung Disease), HTN (Hypertension), HF (Heart Failure), CAD (Coronary Artery Disease), DM (Diabetes Mellitus), HIV (Human Immunodeficiency Virus), and CKD (Chronic Kidney Disease).

### Refugee experiences and mental health stressors use of participants

Among Sudanese refugees with chronic illnesses, the majority of participants had been in the refugee camp for less than a year (119, 51.5%). A significant proportion reported experiencing a shortage of food or clean water (154, 66.7%), smoking cigarettes (137, 59.3%), and inadequate shelter or clothing (156, 67.5%). Additionally, nearly 40% of participants (92, 39.8%) had been imprisoned or detained (Table 2).

**Table 2.** Refugee experiences and mental health stressors of Sudanese refugees with chronic illnesses in Aftit refugee camp, Ethiopia, 2024 (*N* = 231).

Variable	Category	Frequency (%)
Length of stay in refugee camp	<1 year	119 (51.5)
	>1 year	112 (48.9)
Have you chewed Khat in the last three months?	No	122 (52.8)
	Yes	109 (47.2)
Do you currently smoke cigarettes?	No	94 (40.7)
	Yes	137 (59.3)
Have you experienced a shortage of food or clean water in the refugee camp?	No	77 (33.3)
	Yes	154 (66.7)
Have you faced inadequate shelter or a lack of proper clothing?	No	75 (32.5)
	Yes	156 (67.5)
Have any of your family members or friends been killed due to violence?	No	155 (67.1)
	Yes	76 (32.9)
Have you ever been tortured, beaten, or subjected to physical abuse?	No	156 (67.5)
	Yes	75 (32.5)
Have you ever been imprisoned or detained?	No	139 (60.2)
	Yes	92 (39.8)

### Factors associated with depression

**Figure 1.**
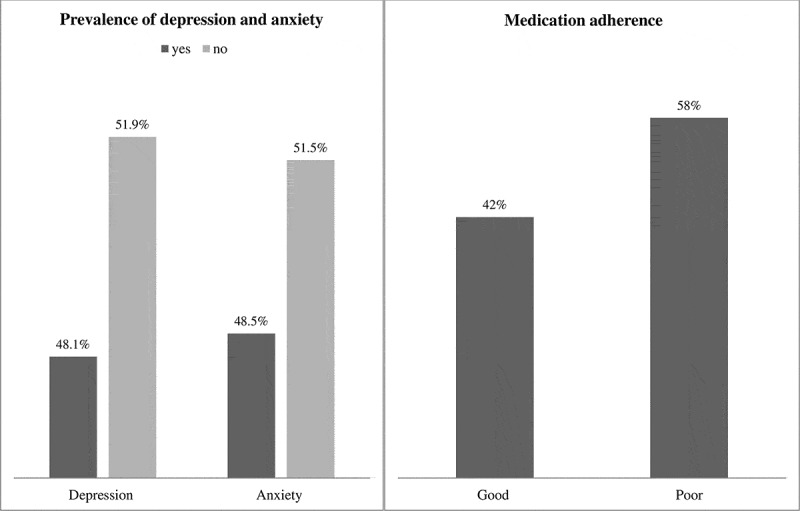
Prevalence of depression, anxiety, and medication adherence among Sudanese refugees with chronic illnesses in Aftit refugee camp, Ethiopia, 2024 (*N* = 231).

Based on our findings, the prevalence of depression among participants in Aftit Refugee Camp is 48.1% (95% CI = 41.6–54.5) (Figure 1). Several important variables are significantly associated with depression symptoms. Among these, living condition was significantly associated with depression. Participants living alone had 6.5 times higher odds of experiencing depression compared to those living with family (AOR = 6.50, 95% CI: 2.85–14.82, *p* < 0.001).Participants who stayed in a refugee camp for one or more years were 3.34 times more likely to experience depression (AOR = 3.34, 95% CI: 1.56–7.17, *p* < 0.002) than those who stayed for less than a year. In addition, Participants who experienced a shortage of food or clean water in the refugee camp were 3.17 times more likely to experience depression (AOR = 3.17, 95% CI: 1.51–6.71, *p* < 0.002) compared to those who did not face such shortages. Similarly, Participants who faced inadequate shelter or a lack of proper clothing were 4.63 times more likely to experience depression (AOR = 4.63, 95% CI: 1.93–11.09, *p* < 0.001) compared to those who had adequate shelter and clothing (Table 3).

**Table 3.** Multivariable logistic regression analysis on factors associated with depression among Sudanese refugees with chronic illnesses in Aftit refugee camp, Ethiopia, 2024 (*N* = 231).

		Depression				
Variables		Yes (%)	No (%)	COR (95% CI)	AOR (95% CI)	*p*-value
Gender	Male	53 (40.5)	78 (59.5)	1	1	
	Female	58 (58.0)	42 (42.0)	2.03 (1.19–3.44)	1.71 (0.79–3.68)	0.170
Marital status	Single	24 (53.3)	21 (46.7)	1	1	
	Married	43 (40.2)	64 (59.8)	0.58 (0.29–1.18)	0.52 (0.21–1.31)	0.168
	Divorced	34 (56.7)	26 (43.3)	1.14 (0.52–2.48)	1.66 (0.61–4.54)	0.320
	Widowed	10 (52.6)	9 (47.4)	0.97 (0.33–2.84)	2.63 (0.59–11.7)	0.204
Living condition	With family	47 (33.3)	94 (66.7)	1	1	
	Alone	64 (71.1)	26 (28.9)	4.92 (2.77–8.74)	6.50 (2.85–14.82)	0.001*
Number of family	1	32 (64.0)	18 (36.0)	1	1	
	2–4	30 (46.9)	34 (53.1)	0.49 (0.23–1.05)	0.95 (0.31–2.92)	0.938
	>4	49 (41.9)	68 (58.1)	0.40 (0.21–0.81)	0.75 (0.27–2.07)	0.586
Types of chronic condition	Resparitory (astma, COPD, Ild)	30 (52.6)	27 (47.4)	1	1	
	Cardiovascular (Htn, HF, CAD)	17 (53.1)	15 (46.9)	1.02 (0.42–2.42)	2.95 (0.91–9.62)	0.073
	Endocrine (DM, thyroide disorder)	13 (43.3)	17 (56.7)	0.68 (0.28–1.67)	1.11 (0.33–3.68)	0.869
	Neurological (Epilepsy, Parkinson)	10 (43.5)	13 (56.5)	0.69 (0.26–1.83)	2.03 (0.57–7.25)	0.273
	Psychatry condition (Depression, Anxity, psychophernia)	29 (54.7)	24 (45.3)	1.08 (0.51–2.31)	2.63 (0.87–7.95)	0.085
	Infectiose (HIV, hepatites)	8 (32.0)	17 (68.0)	0.42 (0.15–1.13)	1.28 (0.33–4.84)	0.716
	Other (onchology, CKD, cirossise … .)	4 (36.4)	7 (63.6)	0.51 (0.13–1.95)	1.48 (0.31–7.11)	0.624
Length of stay in refugee camp	<1 year	44 (37.0)	75 (63.0)	1	1	
	≥1 year	67 (59.8)	45 (40.2)	2.53 (1.49–4.31)	3.34 (1.56–7.17)	0.002*
Have you chewed Khat in the last three months?	No	48 (39.3)	74 (60.7)	1	1	
	Yes	63 (57.8)	46 (42.2)	2.11 (1.24–3.57)	2.49 (1.15–5.41)	0.020*
Do you currently smoke cigarettes?	No	52 (55.3)	42 (44.7)	1	1	
	Yes	59 (43.1)	78 (56.9)	0.61 (0.36–1.03)	0.764 (0.36–1.61)	0.475
Have you experienced a shortage of food or clean water in the refugee camp?	No	20 (26.0)	57 (74.0)	1	1	
	Yes	91 (59.1)	63 (40.9)	4.11 (2.25–7.51)	3.17 (1.51–6.71)	0.002*
Have you faced inadequate shelter or a lack of proper clothing?	No	23 (30.7)	52 (69.3)	1	1	
	Yes	88 (56.4)	68 (43.6)	2.92 (1.63–5.24)	4.63 (1.93–11.09)	0.001*

Abbreviations: COR (Crude Odds Ratio), AOR (Adjusted Odds Ratio), CI (Confidence Interval), COPD (Chronic Obstructive Pulmonary Disease), ILD (Interstitial Lung Disease), HTN (Hypertension), HF (Heart Failure), CAD (Coronary Artery Disease), DM (Diabetes Mellitus), HIV (Human Immunodeficiency Virus), CKD (Chronic Kidney Disease). Hosmer–Lemeshow goodness-of-fit test: *p* = 0.267. *Significance at *p* < 0.05.

### Factors associated with anxiety

The prevalence of anxiety among participants in Aftit Refugee Camp is 48.5% (95% CI = 41.6–55.0) (Figure 1). Age was significantly associated with anxiety;participants aged older than 60 years were 2.98 times more likely to experience anxiety (AOR = 2.98, 95% CI: 1.01–8.82, *p* < 0.048) compared to those younger than 40 years. Marital status also showed a significant association with anxiety. Divorced participants were 5.67 times more likely to experience anxiety (AOR = 5.67, 95% CI: 1.49–21.51, *p* < 0.011), while widowed participants were 8.36 times more likely to experience anxiety (AOR = 8.36, 95% CI: 1.51–46.18, *p* < 0.015) compared to single participants. Additionally, participants who had chewed Khat in the last three months were 2.55 times more likely to experience anxiety (AOR = 2.55, 95% CI: 1.09–5.95, *p* < 0.030) compared to those who had not. Experiencing violence was also significantly associated with anxiety. participants who had a family member or friend killed were 9.68 times more likely to experience anxiety (AOR = 9.68, 95% CI: 4.04–23.20, *p* < 0.001) compared to those who had not. Similarly, participants who had been tortured, beaten, or subjected to physical abuse were 3.37 times more likely to experience anxiety (AOR = 3.37, 95% CI: 1.51–7.50, *p* < 0.003), while those who had been imprisoned or detained were 2.42 times more likely to experience anxiety (AOR = 2.42, 95% CI: 1.01–5.80, *p* < 0.047) compared to those who had not (Table 4).

**Table 4.** Multivariable logistic regression analysis on factors associated with anxiety among Sudanese refugees with chronic illnesses in Aftit refugee camp, Ethiopia, 2024 (*N* = 231).

		Anxiety				
Variables		Yes (%)	No (%)	COR (95% CI)	AOR (95% CI)	*p*-value
Age	<40	19 (25.7)	55 (74.3)	1	1	
	40–60	56 (54.9)	46 (45.1)	3.52 (1.83–6.75)	1.94 (0.77–4.85)	0.155
	>60	37 (67.3)	18 (32.7)	5.95 (2.76–12.82)	2.98 (1.01–8.82)	0.048*
Gender	Male	51 (38.9)	80 (61.1)	1	1	
	Female	61 (61.0)	39 (39.0)	2.45 (1.43–4.18)	1.31 (0.58–2.96)	0.502
Marital status	Single	17 (37.8)	28 (62.2)	1	1	
	Married	47 (43.9)	60 (56.1)	1.29 (0.63–2.63)	1.61 (0.56–4.54)	0.375
	Divorced	38 (63.3)	22 (36.7)	2.84 (1.27–6.32)	5.67 (1.49–21.51)	0.011*
	Widowed	10 (52.6)	9 (47.4)	1.83 (0.61–5.40)	8.36 (1.51–46.18)	0.015*
Educational status	Unable to read and write	31 (54.4)	26 (45.6)	1	1	
	Elementary school	30 (48.4)	32 (51.6)	0.78 (0.38–1.61)	0.83 (0.28–2.45)	0.743
	High school	26 (53.1)	23 (46.9)	0.94 (0.44–2.03)	2.01 (0.63–6.33)	0.234
	Higher institution	25 (39.7)	38 (60.3)	0.55 (0.26–1.14)	1.51 (0.54–4.20)	0.425
Number of family	1	29 (58.0)	21 (42.0)	1	1	
	2–4	31 (48.4)	33 (51.6)	0.68 (0.32–1.43)	1.22 (0.42–3.52)	0.704
	>4	52 (44.4)	65 (55.6)	0.57 (0.29–1.13)	0.44 (0.16–1.18)	0.107
Types of chronic condition	Resparitory (astma, COPD, Ild)	33 (57.9)	24 (42.1)	1	1	
	Cardiovascular (Htn, HF, CAD)	15 (46.9)	17 (53.1)	0.64 (0.26–1.53)	0.38 (0.11–1.31)	0.129
	Endocrine (DM, thyroide disorder)	9 (30.0)	21 (70.0)	0.31 (0.12–0.79)	0.15 (0.33–0.68)	0.014
	Neurological (Epilepsy, Parkinson)	10 (43.5)	13 (56.5)	0.55 (0.21–1.48)	0.54 (0.14–2.05)	0.366
	Psychatry condition (Depression, Anxity, psychophernia)	30 (56.6)	23 (43.4)	0.94 (0.44–2.02)	0.89 (0.33–2.40)	0.820
	Infectiose (HIV, hepatites)	10 (40)	15 (60)	0.48 (0.18–1.26)	0.34 (0.08–1.34)	0.124
	Other (onchology, CKD, cirossise … .)	5 (45.5)	6 (54.5)	0.61 (0.16–2.22)	1.52 (0.27–8.52)	0.633
Number of prescribed medication	1	28 (60.9)	18 (39.1)	1	1	
	2–3	36 (50.0)	36 (50.0)	0.64 (0.31–1.36)	0.85 (0.28–2.52)	0.785
	≥4	48 (42.5)	65 (57.5)	0.47 (0.23–0.95)	0.57 (0.21–1.60)	0.293
Have you chewed Khat in the last three months?	No	50 (41.0)	72 (59.0)	1	1	
	Yes	62 (56.9)	47 (43.1)	1.91 (1.12–3.21)	2.55 (1.09–5.95)	0.030*
Have any of your family members or friends been killed due to violence?	No	53 (34.2)	102 (65.8)	1	1	
	Yes	59 (77.6)	17 (22.4)	6.67 (3.54–12.58)	9.68 (4.04–23.20)	0.001*
Have you ever been tortured, beaten, or subjected to physical abuse?	No	57 (36.5)	99 (63.5)	1	1	
	Yes	55 (73.3)	20 (26.7)	4.77 (2.61–8.76)	3.37 (1.51–7.50)	0.003*
Have you ever been imprisoned or detained?	No	59 (42.4)	80 (57.6)	1	1	
	Yes	53 (57.6)	39 (42.4)	1.84 (1.08–3.14)	2.42 (1.01–5.80)	0.047*

Abbreviations: COR (Crude Odds Ratio), AOR (Adjusted Odds Ratio), CI (Confidence Interval), COPD (Chronic Obstructive Pulmonary Disease), ILD (Interstitial Lung Disease), HTN (Hypertension), HF (Heart Failure), CAD (Coronary Artery Disease), DM (Diabetes Mellitus), HIV (Human Immunodeficiency Virus), CKD (Chronic Kidney Disease). Hosmer–Lemeshow goodness-of-fit test: *p* = 0.325. *Significance at *p* < 0.05.

### Factors associated with medication adherence

**Figure 2.**
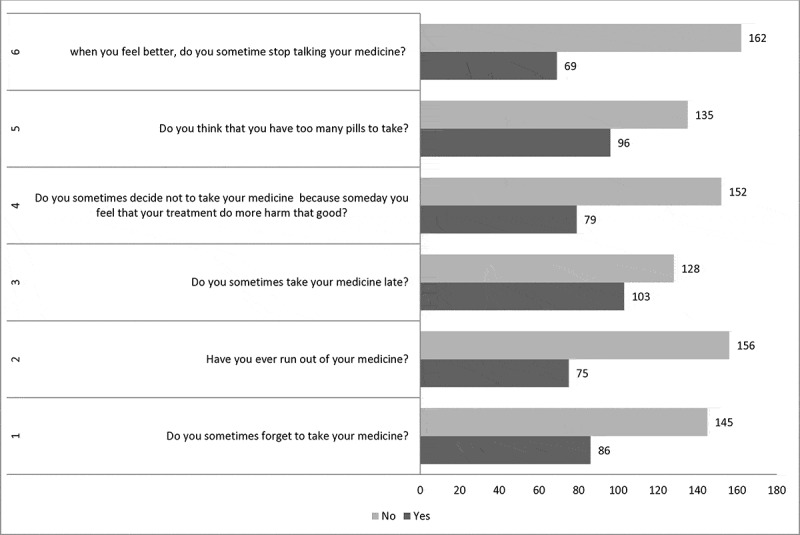
Description of the medication adherence responses among Sudanese refugees with chronic illnesses in Aftit refugee camp, Ethiopia, 2024 (*N* = 231).

Based on our findings, the prevalence of medication adherence among participants in Aftit Refugee Camp is 42% (95% CI = 35.1–48.1) (Figure 1). Several important variables are significantly associated with medication adherence. Age was a significant factor, with participants over 60 years old being 4.78 times more likely to adhere to their medication compared to those under 40 years (AOR = 4.78, 95% CI: 1.54–14.80, *p* = 0.007). Educational status also showed a significant association, as participants who attended a higher institution were 4.88 times more likely to adhere to their medication (AOR = 4.88, 95% CI: 1.77–13.48, *p* < 0.002) compared to those who were unable to read and write. Additionally, living conditions played a role in adherence, with participants living alone being 3.33 times more likely to adhere to their medication (AOR = 3.33, 95% CI: 1.51–7.33, *p* < 0.003) compared to those living with family. Interestingly, participants who had been tortured, beaten, or subjected to physical abuse were significantly less likely to adhere to their medication, with an AOR of 0.16 (95% CI: 0.07–0.40, *p* < 0.001) (Table 5). Description of the medication adherence responses are listed in Figure 2.

**Table 5.** Multivariable logistic regression analysis on factors associated with medication adherence among Sudanese refugees with chronic illnesses in Aftit refugee camp, Ethiopia, 2024 (*N* = 231).

		Adherence				
Variables		Yes (%)	No (%)	COR (95% CI)	AOR (95% CI)	*p*-value
Age	<40	36 (48.6)	38 (51.4)	1	1	
	40–60	38 (37.3)	64 (62.7)	0.62 (0.34–1.15)	2.28 (0.88–5.89)	0.088
	>60	23 (41.8)	32 (58.8)	0.75 (0.37–1.53)	4.78 (1.54–14.80)	0.007*
Marital status	Single	17 (37.8)	28 (62.2)	1	1	
	Married	52 (48.6)	55 (51.4)	1.55 (0.76–3.17)	2.36 (0.91–6.09)	0.076
	Divorced	25 (41.7)	35 (58.3)	1.17 (0.53–2.59)	1.99 (0.67–5.91)	0.213
	Widowed	3 (15.8)	16 (84.2)	0.30 (0.07–1.21)	0.22 (0.04–1.23)	0.087
Educational status	Unable to read and write	20 (35.1)	37 (64.9)	1	1	
	Elementary school	14 (22.6)	48 (77.4)	0.54 (0.24–1.20)	0.59 (0.23–1.54)	0.291
	High school	20 (40.8)	29 (59.2)	1.27 (0.58–2.80)	0.98 (0.35–2.76)	0.974
	Higher institution	43 (68.3)	20 (31.7)	3.97 (1.86–8.50)	4.88 (1.77–13.48)	0.002*
Living condition	With family	47 (33.3)	94 (66.7)	1	1	
	Alone	50 (55.6)	40 (44.4)	2.50 (1.45–4.30)	3.33 (1.51–7.33)	0.003*
Types of chronic condition	Resparitory (astma, COPD, Ild)	27 (47.4)	30 (52.6)	1	1	
	Cardiovascular (Htn, HF, CAD)	10 (31.2)	22 (68.8)	0.50 (0.20–1.25)	1.00 (0.30–3.32)	1.000
	Endocrine (DM, thyroide disorder)	13 (43.3)	17 (56.7)	0.85 (0.34–2.06)	1.42 (0.43–4.63)	0.558
	Neurological (Epilepsy, Parkinson)	7 (30.4)	16 (69.6)	0.48 (0.17–1.36)	0.37 (0.09–1.45)	0.156
	Psychatry condition (Depression, Anxity, psychophernia)	25 (47.2)	28 (52.8)	0.99 (0.46–2.09)	1.71 (0.67–4.35)	0.256
	Infectiose (HIV, hepatites)	7 (28.0)	18 (72.0)	0.43 (0.15–1.19)	0.34 (0.08–1.42)	0.143
	Other (onchology, CKD, cirossise … .)	8 (72.7)	3 (27.3)	2.96 (0.71–12.32)	4.33 (0.66–28.30)	0.125
Number of prescribed medication	1	13 (28.3)	33 (71.7)	1	1	
	2–3	30 (41.7)	42 (58.3)	1.81 (0.81–4.01)	1.42 (0.48–4.23)	0.523
	≥4	54 (47.8)	59 (52.2)	2.32 (1.10–4.87)	2.34 (0.86–6.39)	0.095
Length of stay in refugee camp	<1 year	42 (35.3)	77 (64.7)	1	1	
	>1 year	55 (49.1)	57 (50.9)	1.76 (1.04–2.99)	1.54 (0.73–3.21)	0.251
Have you experienced a shortage of food or clean water in the refugee camp?	No	27 (35.1)	50 (64.9)	1	1	
	Yes	70 (45.5)	84 (54.5)	1.54 (0.87–2.71)	0.74 (0.34–1.58)	0.442
Have any of your family members or friends been killed due to violence?	No	72 (46.5)	83 (53.5)	1	1	
	Yes	25 (32.9)	51 (67.1)	0.56 (0.31–1.01)	0.53 (0.24–1.20)	0.131
Have you ever been tortured, beaten, or subjected to physical abuse?	No	81 (51.9)	75 (48.1)	1	1	
	Yes	16 (21.3)	59 (78.7)	0.25 (0.13–0.47)	0.16 (0.07–0.40)	0.001*

Abbreviations: COR (Crude Odds Ratio), AOR (Adjusted Odds Ratio), CI (Confidence Interval), COPD (Chronic Obstructive Pulmonary Disease), ILD (Interstitial Lung Disease), HTN (Hypertension), HF (Heart Failure), CAD (Coronary Artery Disease), DM (Diabetes Mellitus), HIV (Human Immunodeficiency Virus), CKD (Chronic Kidney Disease). Hosmer–Lemeshow goodness-of-fit test: *p* = 0.768. *Significance at *p* < 0.05.

## Discussion

This study found that nearly half of the Sudan Refugees with chronic illness at Aftit Refugee Camp experienced symptoms of depression (48.1%, 95% CI = 41.6–54.5) and anxiety (48.5%, 95% CI = 41.6–55.0). The prevalence of depression in our study is in line with a study done on Eritrea refugees at 45% [29], in southern Sudan at 49.9% [30], in Uganda at 46.2% [31], in Karenina Refugees at 41.8% [32], and in Syria refuge at 47.7% [33]. However, this is higher than the study done in northern Uganda at 15.2% [34], Somali refugees in Kenya at 40.8% [28], Greek at 33.3% [35], and Turkey at 34.7% [36]. These differences may reflect variations in study populations, as our research focused on refugees with chronic illnesses requiring medication, where the added burden of disease management likely heightens vulnerability to depression and anxiety [37]. Additionally, variations in depression assessment tools, access to mental health services, and the political and social environment may also explain these differences [38]. Prolonged displacement and uncertainty about the future can further impact mental well-being. Nevertheless, our study finding is lower than the study conducted in Somalia at 59% [39], in South Africa at 54.6% [40], Syrian refugees at 59.4% [41], and Gulu and Amuru districts of northern Uganda at 67% [42], which may be explained by differences in conflict exposure, trauma histories, and post-migration stressors.

Similarly, the anxiety prevalence observed in this study is consistent with findings from South Africa (49.4%) [40], in Somalia at 43.7% [43], in Karenni refugees in Thai – Burmese at 41% [32], in the USA at 40.3% [33]. However, this is higher than the study done on Syrian refugees at 13.5% [44], Australians at 20% [45], in Germany Arabic speaking refugees at 26.8% [46]. This deference may be due to the psychological impact of separation anxiety, combined with the stress of adjusting to a new life and the challenges of relocation, such as adapting to a new culture, finding stability, and building new relationships [47]. Additionally, differences in study design such as a three-year follow-up period in Germany, the use of the DASS-21 tool, and a smaller sample size (148 participants) in an Australian study might also help explain these results. Nevertheless, our study finding is lower than the study conducted in Uganda at 73% [48], in Bangladesh at 70% [49], Afghanistan at 72.2% [50]. Where access to healthcare, stigma, cultural perceptions of mental health, and camp conditions may intensify the risk [51].

Additionally, this study identified factors significantly associated with depression; the strongest factor associated with depression in this study was living alone. Refugees with chronic illnesses who live alone have 6.5 times greater chances of experiencing depressive symptoms compared to those living with family. This might be a result of the loneliness and isolation that come with living alone. It can be considerably more difficult for people to deal with the difficulties of chronic illness without the support of family, which frequently results in emotions of hopelessness and despair [52]. Additionally, those who live alone may also have a harder time getting access to mental health and medical care. On the other hand, people who live with family usually benefit from shared responsibilities and emotional support, helping to reduce stress and improve mental health [53]. This highlights how crucial it is to provide refugees with chronic illnesses with appropriate living arrangements, as this can significantly impact their general well-being. Our research findings align with evidence from systematic reviews and meta-analyses that assess the link between living alone and an increased risk of depression in longitudinal studies [54].

Another variable significantly associated with depression is who experience a shortage of food; clean water, shelter, and clothing were more likely to experience depression than those who do not. This may be because a lack of clothing, food, clean water, and shelter causes a great deal of tension and anxiety [55]. Refugees who are continuously concerned about accessing their basic needs may experience pessimism and despair, which frequently contribute to depression. Furthermore, living in hazardous conditions and not eating enough can hurt one’s physical and mental well-being [56]. It should come as no surprise that migrants with chronic illnesses are more prone to suffer from depressive symptoms when you consider these physical difficulties with the psychological stress of uncertainty [57]. Additional important variables significantly associated with depression symptoms are the length of stay in refugee camps. Refugees with chronic illnesses who lived in one or more than a year in a refugee camp were 3.34 times more likely to experience depressive symptoms compared to those living with family. This may be because people who live longer in refugee camps experience greater stress and uncertainty [58]. Poor living conditions, limited healthcare access, and uncertainty about the future can worsen mental health, leading to hopelessness and isolation, especially in those with chronic illness. These factors together may explain the higher prevalence of depression, consistent with other studies [37,59].

Another significant associate variable is Khat chewing; refugees with chronic illnesses who chew Khat are 2.49 times more likely to experience depression than those who do not. This could be because of the psychotropic effects of Khat, which can alter a person’s mood and behavior. Long-term use of Khat can exacerbate feelings of restlessness, anxiety, and depression, even though some individuals chew it to help them deal with stress or worry [60]. Furthermore, for refugees who already have chronic illnesses, the social and financial difficulties associated with Khat use, such as financial difficulties or social isolation, can exacerbate mental health problems, creating a vicious cycle in which reliance on Khat can exacerbate mental health [60].

The strongest factor linked to anxiety was exposure to trauma, as refugees who experienced murder of family or friends, torture, or imprisonment were significantly more likely to develop anxiety. This may be because of psychological trauma caused by these events, leading to persistent fear, grief, and emotional distress. Such experiences can trigger post-traumatic stress, intrusive thoughts, and a constant sense of insecurity, making it difficult to cope with daily life [61]. Additionally, the lack of mental health support in refugee camps may leave individuals struggling to process their trauma, further worsening anxiety [62].

An additional strong variable significantly associated with anxiety symptoms is marital status. Refugees with chronic illnesses who were divorced or widowed were more likely to experience anxiety symptoms compared to single individuals. This may be because losing a spouse results in the loss of a social and emotional support system, which can induce intense emotions of grief and loneliness. The difficulties of everyday life might seem even more daunting when one is living alone in a refugee camp, without a companion to share duties or provide solace. Their stress levels may rise as a result of financial difficulties, restricted access to treatment, and future uncertainty. Our study finding is supported by other studies done on Syrian refugee women in Jordan [63].

Another factor was age, with refugees over 60 being 2.49 times more likely to experience anxiety than those under 40. The reason for this may be due to various factors, including heightened health concerns, social isolation, and the stress of living in a refugee camp [64]. Older individuals often face significant challenges in managing their chronic illnesses and adapting to their environment, which can lead to increased anxiety [65]. Furthermore, the loss of social support and uncertainty about the future can weigh heavily on their minds. This result is supported by research published in The American Journal of Geriatric Psychiatry [66]. Another significant associate variable is Khat chewing; refugees with chronic illnesses who chew Khat are 2.55 times more likely to experience anxiety than those who do not. The stimulating effects of Khat may be the cause, as they can exacerbate anxiety symptoms by making people feel tense, restless, and agitated [67]. Regular use can eventually result in dependence, and stopping use can cause tension and mood swings. Additionally, because some people may put Khat chewing above other important demands, it is frequently linked to financial difficulties and familial conflicts. They may find it even more difficult to cope with their chronic illnesses and day-to-day struggles in the camp as a result of this cycle of stress and anxiety [68].

This study reveals that 42% (95% CI = 35.1–48.1) of participants at Aftit Refugee Camp had good medication adherence. The findings of this study are higher than those of a study conducted among refugees in Zaatari camp, Jordan, where 22.8% of refugees had good adherent [11], in Syria at 21.8% of refugees had good adherent [69], Palestine refugees, where 27% of refugee had good adherent [70]. However, it’s lower than the study done in Lebanon at 70% were moderately to highly adherent [71], and in USA 75% had good medication adherence [72]. The reason for this discrepancy may be due to the differences in healthcare access, the availability of medications, and the educational status of refugees [73]. Refugees in Lebanon and the USA may have better healthcare resources and easier access to medications than Zaatari Camp, Syria, and Palestine refugees who could help them stick to their treatment plans. Additionally, differences in how the studies were conducted, including their methodologies, the populations they focused on, and how adherence was defined, could also explain some of the variation in findings [11].

In our study, participants aged over 60 were 4.78 times more likely to stick to their medication compared to those under 40. This may be because older individuals are more aware of the importance of managing their health and the risks of complications. They might also feel a stronger responsibility to take care of themselves due to past experiences with illness. Plus, older refugees often have more consistent follow-ups with healthcare providers [74]. Another significant associated variable among participants is having completed higher education and living alone. The reason may be due to people with higher education may have a better awareness of their health and the importance of keeping to their prescription regimen, which can lead to improved adherence [75]. Refugees who live alone may have greater control over their healthcare decisions, making them feel more accountable for maintaining their health on their own [75]. The other negatively associated variable is participants who have experienced tortured, beaten, or subjected to physical abuse were less likely to adhere to their medication. The reason may be that trauma and abuse can lead to psychological distress, including anxiety, depression, and post-traumatic stress disorder (PTSD), which can affect their ability to prioritize and manage their health. Additionally, these experiences may diminish trust in healthcare systems, reduce the sense of self-efficacy, and create emotional barriers that hinder medication adherence [76].

This study has several limitations that should be considered when interpreting the findings. First, the cross-sectional design captures data at a single point in time and therefore does not allow for causal inferences. Second, reliance on self-reported measures may introduce reporting bias, as participants could overstate adherence to appear more compliant. Third, the generalizability of the results is limited because the sample was drawn from a single refugee camp and may not represent other refugee or displaced populations. Future studies employing longitudinal or case-control designs, and including diverse settings, would provide a deeper understanding of these associations.

## Conclusion

Addressing the mental health and well-being of refugees with chronic illnesses requires attention not only to their medical needs but also to the social and environmental challenges they face. This study recommends routine screening and targeted support for refugee participants with chronic illnesses, particularly for those at higher risk of depression and anxiety. Special attention should be given to older individuals, those living alone or divorced, refugees who have stayed in camps for more than a year, substance users such as Khat users, and individuals who have experienced food and shelter shortages, torture, or imprisonment. Based on the findings of our results, it’s essential to integrate mental health support into routine care for refugees with chronic illnesses. Healthcare providers should be trained to recognize and address depression and anxiety, ensuring that emotional well-being is considered alongside physical health. Enhancing psychosocial support within refugee health programs may provide meaningful benefits in addressing these challenges. Efforts to improve medication adherence should focus on patient education, peer support, and culturally sensitive counseling. Beyond medical care, providing stable housing, food security, and support for those struggling with substance use can make a real difference in improving both mental health and overall well-being for refugees facing these challenges. Future research across multiple camps or countries would help to better understand these dynamics and guide broader interventions and policies.

## Supplementary Material

STROBE.doc

## Data Availability

All data are in the manuscript; additional data are available on request.
