# Supplementary material for: Depression, anxiety, and medication adherence among Sudanese refugees with chronic illnesses in Aftit refugee camp, northwest Ethiopia: a cross-sectional study
Source: Glob Health Action. 2025 Oct 29;18(1):2573509. doi: 10.1080/16549716.2025.2573509 (PMC12573532; doi:10.1080/16549716.2025.2573509)
Supplement: STROBE.doc [file ZGHA_A_2573509_SM9663.doc]

STROBE Statement—Checklist of items that should be included in reports of ***cross-sectional studies***

|  | Item No | Recommendation |
| --- | --- | --- |
| **Title and abstract** | 1 | (a)The study is identified as a “cross-sectional study” in the abstract. Page 2 |
|  |  | (*b*) The abstract provides a concise summary of background, objectives, methods, and main findings. Page 2 |
| Introduction | | |
| Background/rationale | 2 | Well-developed background explaining the Sudan conflict, refugee crisis, and rationale for investigating depression, anxiety, and medication adherence. section 1 | Yes, Page 3 |
| Objectives | 3 | Stated clearly: To assess the prevalence and associated factors of anxiety, depression, and medication adherence among Sudanese refugees with chronic diseases, last paragraph, in the abstract | Yes, page 4 |
| Methods | | |
| Study design | 4 | Cross-sectional design is mentioned early in both abstract and methods. Abstract and section 2.1 | Yes, page 4 |
| Setting | 5 | Study took place at Aftit refugee camp, Ethiopia, between October 1–30, 2024. Abstract and section 2., Page 4 and 5 |
| Participants | 6 | Sudanese refugees aged ≥18 years, taking chronic medications for ≥3 months. Random sampling used. Page 5 |
| Variables | 7 | Clearly defined dependent (anxiety, depression, adherence) and independent variables (age, gender, education, trauma, etc.). Sections 2.5 (Variables) and 2.8 (Operational definitions), page 6 and 8 |
| Data sources/ measurement | 8* | HSCL-25 used for anxiety/depression, 6-item tool for adherence. Validated Arabic version used. Section 2.6 (Data collection tools and procedures). Page 7 |
| Bias | 9 | Discussed social desirability/self-report bias in the limitations. Section 2.7 (Data quality control). Page 7 and 25 |
| Study size | 10 | Sample size calculated using standard formula, adjusted with finite population correction. Final n = 240; 231 completed. Section 2.4 (Sample size calculation). Page 5 and 6 |
| Quantitative variables | 11 | Grouped meaningfully (e.g., age categories, number of medications). Explained in tables and methods. Page 10 and 11 |
| Statistical methods | 12 | (*a*) Bivariate and multivariate logistic regression described. |
|  |  | (*b*) Subgroups and confounders handled in models. |
|  |  | (*c*) There were no missing data. All participant include the analysis had complete response. |
|  |  | (*d*) No explicit mention of stratified analysis. |
|  |  | (*e*) No sensitivity analysis conducted. |
| Results | | |
| Participants | 13* | (a) 231 out of 240 responded (96.25%). Page 10 |
|  |  | (b) Refused 5 and Incomplete response 3. Page 10 |
|  |  | (c) Not applicable |
| Descriptive data | 14* | (a) Detailed tables and text provide participant characteristics. |
|  |  | (b) Missing data per variable not reported |
| Outcome data | 15* | Prevalence rates for depression (48.1%), anxiety (48.5%), and adherence (42%) with 95% CI. Page 12, 15 and 18 |
| Main results | 16 | (*a*) AORs, 95% CI reported. Confounders discussed. From page 10-20 |
|  |  | (*b*) Categorization of variables (e.g., age, adherence) explained. |
|  |  | (*c*) Absolute risks not relevant here. |
| Other analyses | 17 | No sensitivity analysis |
| Discussion | | |
| Key results | 18 | Summarized in the discussion with reference to objectives. Page 21-35 |
| Limitations | 19 | Cross-sectional design, generalizability, and self-report bias all acknowledged. |
| Interpretation | 20 | Cautious interpretation supported by comparisons with literature. |
| Generalisability | 21 | Applicability to similar low-resource refugee settings briefly discussed. |
| Other information | | |
| Funding | 22 | Clearly stated: “Not funded.” Page 27 |

*Give information separately for exposed and unexposed groups.

**Note:** An Explanation and Elaboration article discusses each checklist item and gives methodological background and published examples of transparent reporting. The STROBE checklist is best used in conjunction with this article (freely available on the Web sites of PLoS Medicine at http://www.plosmedicine.org/, Annals of Internal Medicine at http://www.annals.org/, and Epidemiology at http://www.epidem.com/). Information on the STROBE Initiative is available at www.strobe-statement.org.
